# Prognostic and clinicopathological value of FoxM1 expression in colorectal cancer: A systematic review and meta-analysis

**DOI:** 10.1097/MD.0000000000013899

**Published:** 2018-12-28

**Authors:** Yizhou Yao, Xuchao Wang, Linhua Jiang, Xinyu Shao, Xinguo Zhu, Songbing He

**Affiliations:** a Department of General Surgery, The First Affiliated Hospital of Soochow University; b Department of Gastroenterology, The Affiliated Suzhou Hospital of Nanjing Medical University, Suzhou, Jiangsu, China.

**Keywords:** colorectal cancer, FoxM1, meta-analysis, prognosis, systematic review

## Abstract

**Background:**

The study aims to assess the relationship between FoxM1 expression and clinicopathological parameters and prognosis of patients diagnosed with colorectal cancer (CRC) by summarizing the studies included.

**Methods:**

PubMed, EMBASE, The Cochrane Library and other sources were searched for relative studies. Odds ratio (OR) and confidence interval (CI) were used to assess association between FoxM1 expression and clinical parameters and prognosis of CRC patients.

**Results:**

Eight studies were included in the final analysis, with 1149 CRC patients. The outcome revealed that expression of FoxM1 was associated with lymph node metastasis (OR = 0.33, 95%CI = 0.19–0.62, *P < *.001), distant metastasis (OR = 0.35, 95%CI = 0.24–0.46, *P < *.001) and tumor node metastasis (TNM) stage (OR = 0.45, 95%CI = 0.29–0.72, *P < *.001). Meanwhile, reduced FoxM1 expression indicated higher 5-year survival rate (OR = 0.38, 95%CI = 0.18–0.78, *P* = .01). Expression of FoxM1 was also increased obviously in CRC tissues (OR = 13.04, 95%CI = 4.07–41.71, *P < *.001).

**Conclusion:**

This pooled analysis indicated that FoxM1 expression related to lymph node metastasis, distant metastasis, TNM stage and poor prognosis of the CRC patients.

## 1 Introduction

Colorectal cancer (CRC) is the third most common cancer and the third leading cause of cancer death in the United States.^[1]^ In recent years, although the great development of detection technology, surgical skills, and neoadjuvant chemotherapy, the prognosis of patients with advanced CRC remained poor. Especially in China, the 5-year survival rate of the advanced CRC patients is only 8%.^[2]^ Due to the large population, the situation is more serious in China. Therefore, there is an urgent need to investigate the molecular mechanisms of CRC and identify new biomarkers for early diagnosis and targeted treatment. Patients who have lost the opportunity of radical surgery will benefit from new treatment strategies.

Forkhead box M1 (FoxM1), one of the FOX family of transcription factors, plays multiple roles in cell proliferation and metabolism, such as regulating the cell cycle transition from the G1 to the S phase, as well as the progression to mitosis.^[3–5]^ Aberrant activation of FoxM1 relates to tumorigenesis and progression of several kinds of malignancies, including liver, breast, prostate, brain, and lung cancer.^[6–9]^ However, the function of FoxM1 in the CRC has still not been elucidated clearly. In the studies published, the relationship between the FoxM1 expression level and clinical parameters and survival has been investigated.^[10,11]^ Whereas, the results of these studies were inconsistent. Considering the controversies of current findings, we undertook a meta-analysis with all eligible studies to assess the potential relationship between the expression of FoxM1 and clinical pathological parameters and survival of CRC patients. The results showed supportive evidence that FoxM1 could be an effective therapeutic target in CRC.

## 2 Materials and methods

This meta-analysis was conducted according to the Preferred Reporting Items for Systematic Reviews and Meta-Analysis (PRISMA) guidelines.^[12]^ Because the studies included in this meta-analysis have been published, the ethical approval from ethics committees was not needed.

### 2.1 Search strategy

PubMed, EMBASE, The Cochrane Library, Elsevier, Web of Science and other sources were searched. The search was performed by using the keywords including ("CRC” or "colon cancer” or "rectal cancer” or "colorectal neoplasms” or "rectal carcinoma”) and ("survival” or "prognosis”) and ("FoxM1”). These keywords were searched alone or in combination without limitation for language. The eligible articles were also identified by scanning all potentially relevant studies and their references. The latest search was done on April 2018.

### 2.2 Selection criteria

Studies meeting the following criteria were considered eligible to be included in this meta-analysis:

1. the patients diagnosed with CRC;
2. the relationship between expression of FoxM1 and clinical parameters or survival of patients with CRC investigated;
3. original research;
4. only studies assessed identical target factors included.

The exclusion criteria were as follows:

1. animal research;
2. the literatures without relatively complete research data and specific number of patients;
3. repeated studies or the same database or patients;
4. Survival of patients assessed with different treatment.

### 2.3 Data extraction

Eligible trial selection was performed independently by 2 authors. In addition, 2 researchers independently assessed the data of the first author, publication date, research design, patients (number, characteristics), study period, sample size, gender, differentiation of cancer, depth of invasion, lymph node metastasis, tumor node metastasis (TNM) stage and overall survival (OS). Any disagreements proposed were discussed with a third author to reach a consensus by analyzing the original data again. The Cochrane Collaboration Risk of Bias Tool was used to assess quality of the included studies.

### 2.4 Statistical analysis

The meta-analysis was performed using the software of Review Manager 5.3. The results were presented as odds ratio (OR) with 95%CI for clinical outcomes. Heterogeneity in the trials was performed by using chi-squared test. Primary analyses were done with a fixed effects model when *P*_heterogeneity_ ≥ .1 or *I*^2^ < 50%. If considerable heterogeneity was found (*P*_heterogeneity_ ≤ .1 or *I*^2^ > 50%), further analysis was done with a random effects model. *P < *.05 for both tests was considered statistically significant. The software of Stata 12.0 (STATA, College Station, TX, USA) and RevMan 5.3 software (Cochrane Collaboration) were used for the analysis.

## 3 Results

### 3.1 Literature search and study characteristics

**Figure 1:**
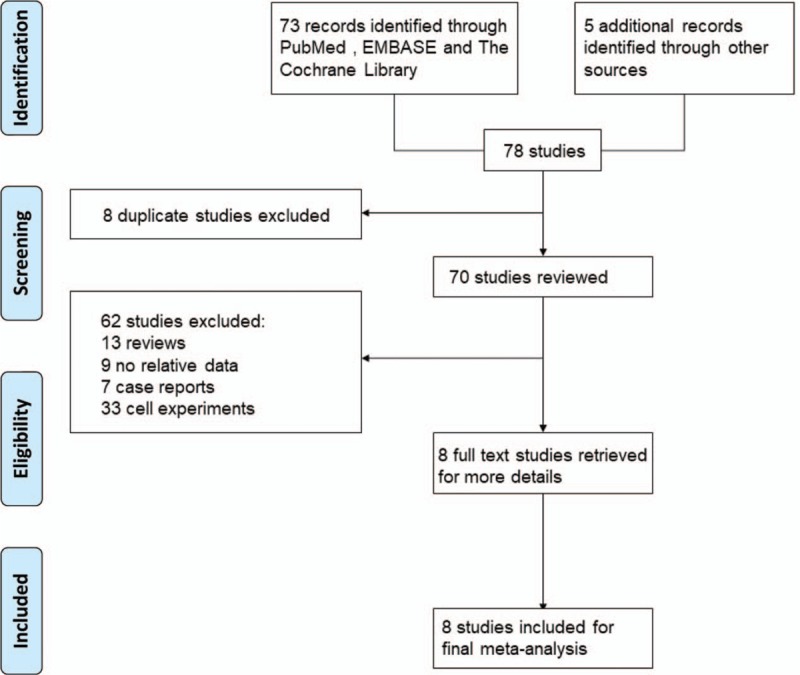
Flow diagram of study selection.

Figure 1 showed a flow diagram of the selection process for the studies included in the final analysis. Initially, 78 references were identified from PubMed, EMBASE, The Cochrane Library, Elsevier, Web of Science and other sources. We screened these articles. Eight records data were repeated from the same population and were eliminated. Then in the rest 70 articles, 62 articles were excluded because of review only (N = 13), no relative data (N = 9), case reports (N = 7), and cell experiments (N = 33). Eight trials were included in the final analysis.^[10–17]^ A total of 1149 patients were included in this meta-analysis. Table 1 summed up the basic characteristics of the included studies.

**Table 1:**
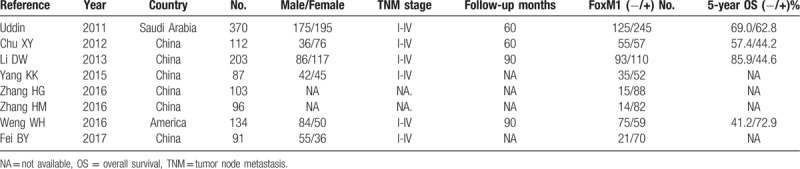
Characteristics of included studies.

**Figure 2:**
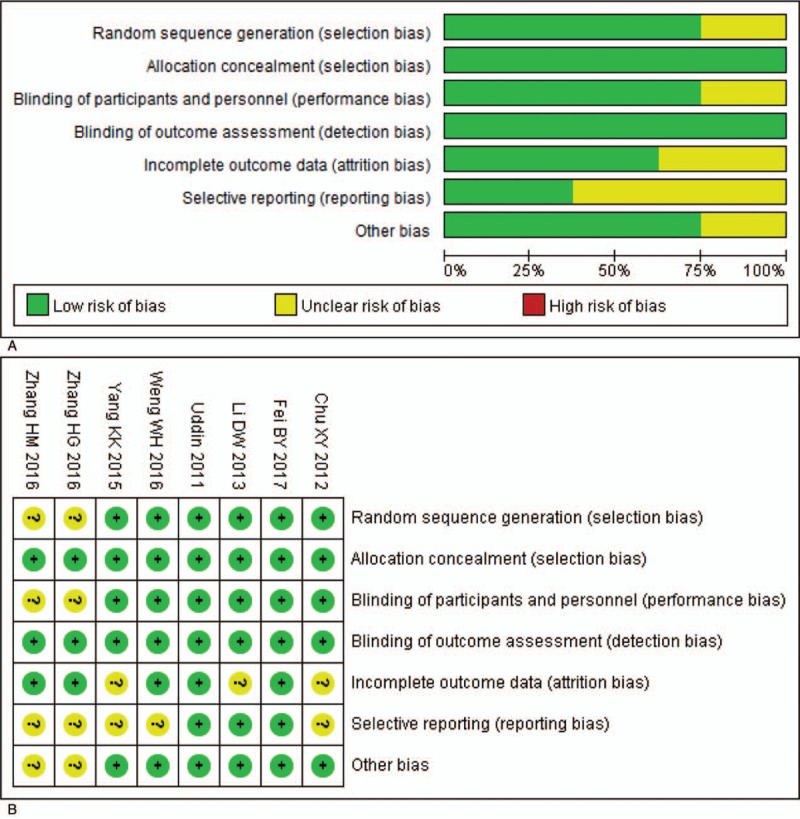
Assessment of risk of bias. A: Methodological quality graph: authors’ judgment about each methodological quality item presented as percentages across all included studies; B: Methodological quality summary: authors’ judgment about each methodological quality item for each included study, "+” low risk of bias; "?” unclear risk of bias; "-” high risk of bias.

Quality assessment indicated that allocation concealment and blinding of outcome assessment were low in all the included studies. Two studies carried out by Fei et al^[16]^ and Uddin et al^[11]^ had a relatively high quality (Fig. 2A and B).

### 3.2 Correlations of FoxM1 expression with clinicopathological characteristics

**Figure 3:**
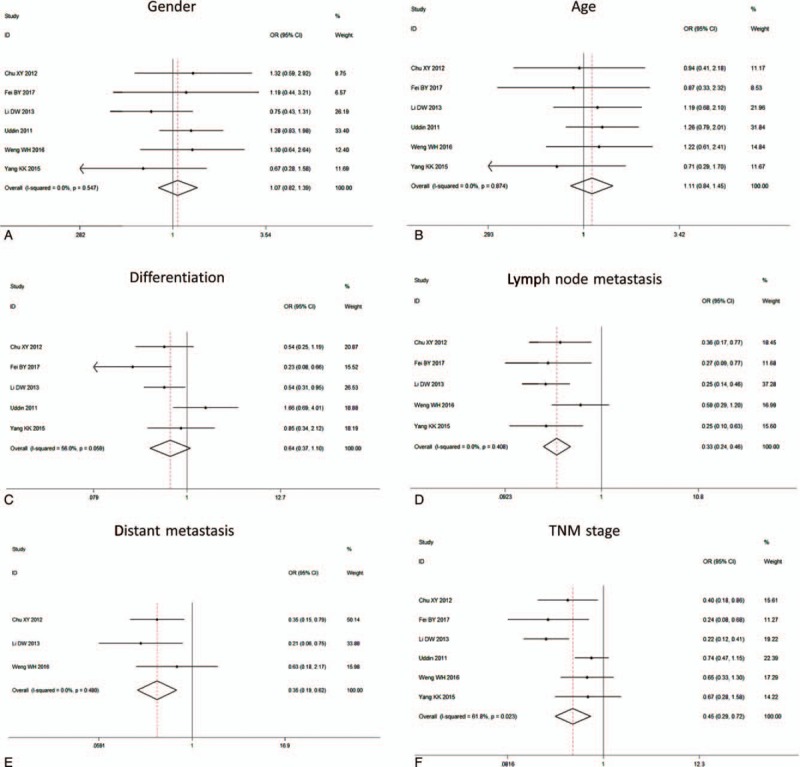
The forest plot of ORs for the association between FoxM1 expression and the A: gender, B: age, C: differentiation, D: lymph node metastasis, E: distant metastasis, F: TNM stage. FoxM1 = Forkhead box M1, OR = odds ratio, TNM = tumor node metastasis.

Results revealed that FoxM1 expression was not associated with the gender (OR = 1.07, 95%CI = 0.82–1.39, *P* = .61, fixed effect), age (OR = 1.11, 95%CI = 0.84–1.45, *P* = .46, fixed effect) and differentiation (OR = 0.64, 95%CI = 0.37–1.10, *P* = .11, random effect) in CRC patients (Fig. 3A–C, Table 2). However, FoxM1 expression had a remarkably close link with lymph node metastasis (OR = 0.33, 95%CI = 0.19–0.62, *P < *.001, fixed effect), distant metastasis (OR = 0.35, 95%CI = 0.24–0.46, *P < *.001, fixed effect) and TNM stage (OR = 0.45, 95%CI = 0.29–0.72, *P < *.001, random effect) (Fig. 3D–F).

**Table 2:**
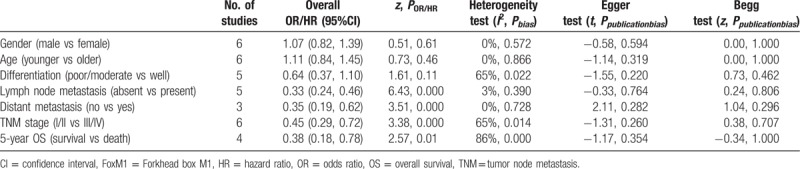
Main results for meta-analysis between FoxM1 and clinicopathological factors.

### 3.3 Correlation of FoxM1 expression with overall survival

**Figure 4:**
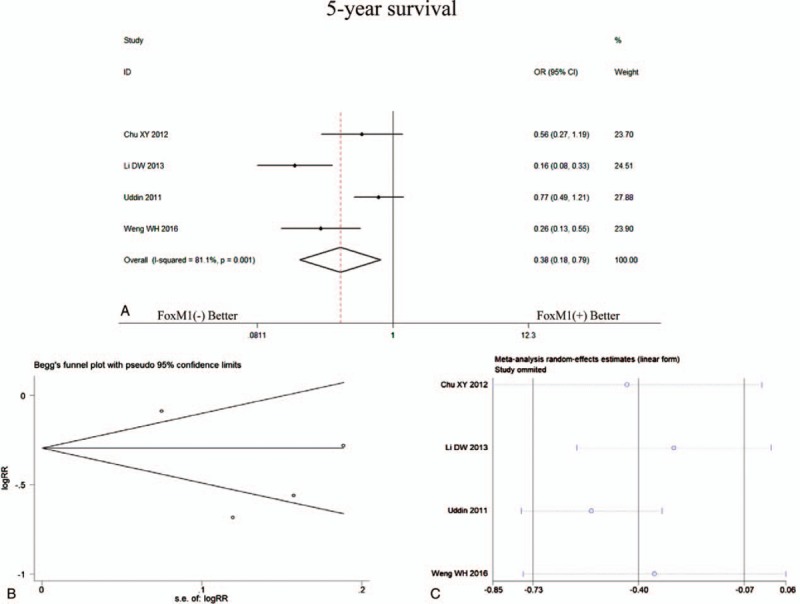
The forest plot showed the analysis for 5-year survival of CRC patients according to FoxM1 expression (A), funnel plots for publication bias (B) and sensitivity analysis (C) of comparisons between expression of FoxM1 and 5-year survival. CRC = colorectal cancer, FoxM1 = Forkhead box M1.

First, we analyzed 5 studies included and found an obvious difference between the expression of FoxM1 in CRC tissue and paired adjacent normal colorectal tissue (OR = 13.04, 95%CI = 4.07–41.71, *P* < .001). Then, the association between FoxM1 expression and prognosis was analyzed. The related data was acquired from four included studies directly or extracted from the Kaplan–Meier survival cure. Our meta-analysis indicated that there were statistical associations between FoxM1 expression and the poor 5-year survival (OR = 0.38, 95%CI = 0.18–0.78, *P* = .01, random effect) (Fig. 4A).

### 3.4 Publication bias and sensitivity analysis

**Figure 5:**
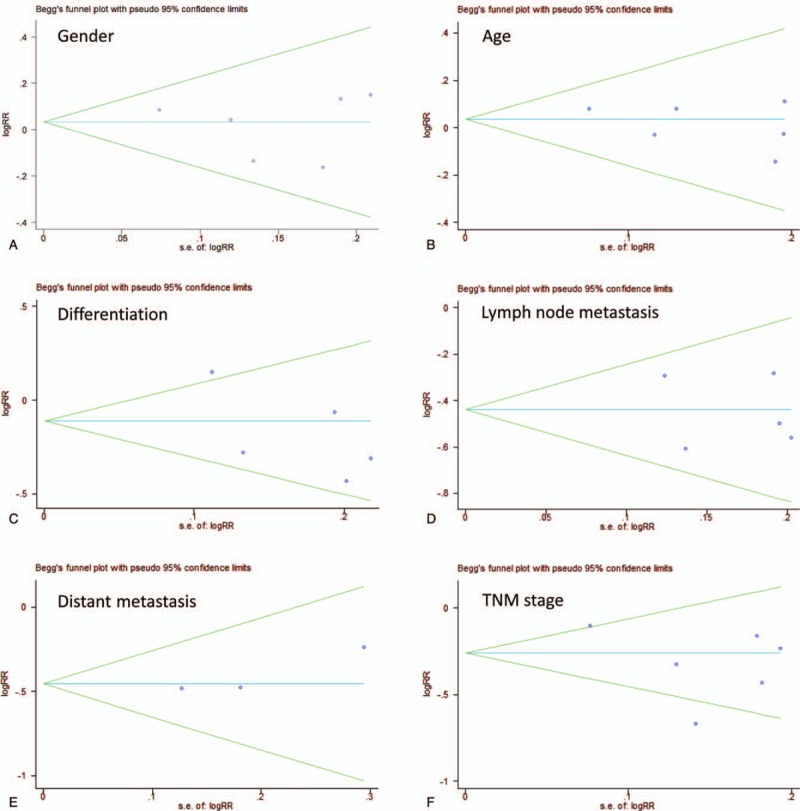
Funnel plots for publication bias. A: gender, B: age, C: differentiation, D: lymph node metastasis, E: distant metastasis, F: TNM stage. TNM = tumor node metastasis.

**Figure 6:**
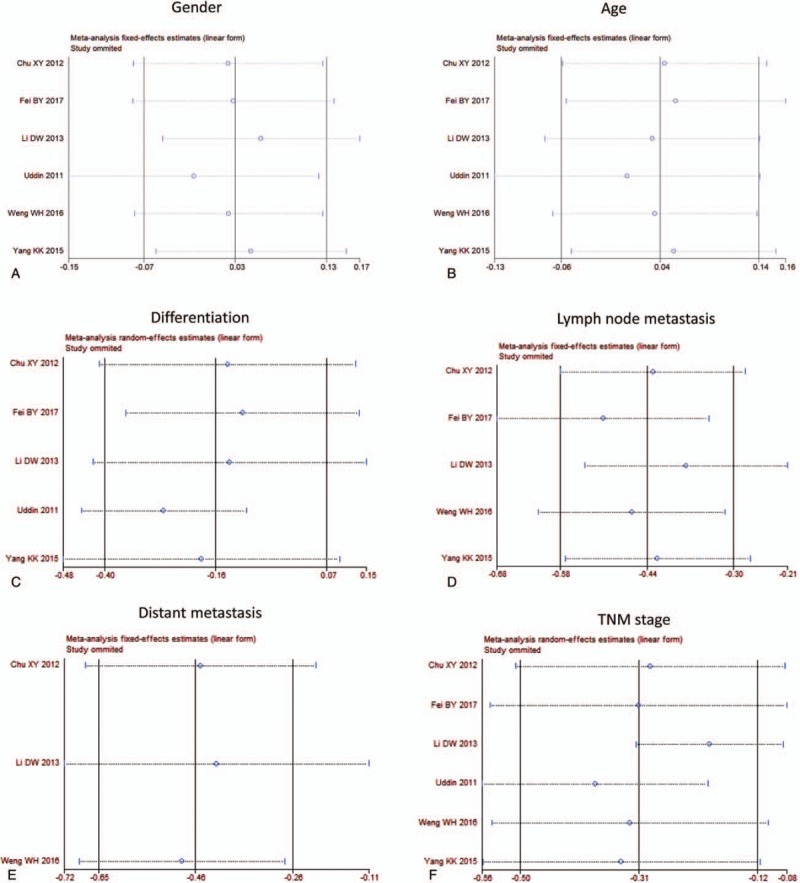
Sensitivity analysis. A: gender, B: age, C: differentiation, D: lymph node metastasis, E: distant metastasis, F: TNM stage. TNM = tumor node metastasis.

None of the significant publication bias was indicated by Egger or Begg tests in 5-year survival analysis (Fig. 4B) and clinicopathological characteristics (Fig. 5A–F, Table 1). Sensitivity analyses were also performed, and it suggested that there was no significant change in pooled OR of survival (Fig. 4C) and clinicopathological characteristics (Fig. 6A–F).

## 4 Discussion

In recent years, the development of surgical skill, neoadjuvant chemotherapy and the novel therapeutic strategies of various agents in the realm of CRC treatment has been great. But many patients were still diagnosed with CRC in an advanced stage or metastasis stage, which indicated poor prognosis. Some molecular markers have been identified to be close to the carcinogenesis of CRC. For example, APC promoter methylation, SMAD4, and CXCR4 could be a biomarker for the diagnosis and prognosis of CRC.^[18]^ Thus, finding more effective biomarkers for early diagnosis and targeted treatment is absolutely essential in CRC related fields.

FoxM1 is regarded as an oncogenic transcription factor and aberrant activation of FoxM1 is considered to be associated with the proliferation and metastasis of human CRC cells.^[10–12,16]^ Some studies reported that FoxM1 might act as an independent role in diagnosis and prognosis of CRC.^[19,20]^ However, individual trials were limited to insufficient specimens and different experimental environments. A further analysis is needed to probe into whether the abnormal increase of FoxM1 expression correlated with the occurrence of CRC and indicated poor survival.

Relative research considered FoxM1 played an essential role in cell proliferation and progression of the cell cycle. Meanwhile, in many kinds of tumors, it was involved in the processes of cell migration, invasion, drug-resistance.^[21–23]^ In CRC, several studies investigated the relationship between the expression of FoxM1 and gender, age, differentiation, lymph node metastasis, and TNM stage and the outcomes were inconsistent. Thus, it is necessary to carry out a pooled study to evaluate the association between FoxM1 and CRC.

In this meta-analysis, we did not observe significant differences in FoxM1expression with gender and age of patients. In differentiation analysis, there was a modest decrease of FoxM1 expression in well-moderate differentiated group. But it did not reach a significant difference. Li et al and Fei et al considered FoxM1 expression was aberrant in poor differentiated CRC tissue,^[10,16]^ but other three studies thought they had no correlation. In addition, the pooled analysis showed that FoxM1 expression was obviously associated with lymph node metastasis, distant metastasis and TNM stage in CRC.

In many kinds of tumor, high expression of FoxM1 indicated poor survival.^[24–26]^ Some pooled analysis also supports the relationship between high expression of FoxM1 and poor OS in patients.

In the GSE dataset, FoxM1-high expression showed weaker tendency to be associated with poor OS (*P* = .397).^[17,27]^ However, FoxM1-high expression group significantly linked with poor OS (*P* = .017) in the TCGA dataset.^[28,29]^ In this CRC meta-analysis, we observed that high expression of FoxM1 was associated with a poor 5-year survival rate. Moreover, we also analyzed the expression of FoxM1 in CRC tissue and paired adjacent normal colorectal tissue. And the FoxM1 expression level increased obviously in CRC tissue. The result was similar to the trend of TCGA database.

There are some limitations should be acknowledged in this meta-analysis. Firstly, the number of studies and total patients included is small. Besides, we cannot get the first-hand individual patient information to verify its accuracy. Secondly, most of the patients included in these studies came from Asia, especially from China, which resulted in certain regional limitations. Thirdly, retrospective studies comprised the majority instead of prospective studies and many studies preferred to report the positive results. Fourthly, Immunohistochemical staining of FoxM1 was detected using a semiquantitative scoring system for both staining intensity and the percentage of positive cells. Most of the studies detected the FoxM1expression by IHC, the antibody types, the cut-off values and the fixation method for paraffin-embedded tissues were diverse, which might cause some biases in pooled analysis. Finally, some studies did not provide OR and 95%CI directly. We extracted OR and 95%CI from the Kaplan–Meier survival curves. However, the method of extrapolating OR from survival curves may be less accurate, which might generate an influence on the outcome. Therefore, our estimation of the function of FoxM1 in CRC may have been overestimated. Further studies are needed to validate the association between FoxM1 expression and CRC related clinicopathological parameters and survival of CRC patients, which supplies a possible option for the anti-FoxM1 treatment for CRC.

In conclusion, comparing the FoxM1 expression and clinicopathologic parameters of CRC patients indicates that the expression of FoxM1 is associated with lymph node metastasis, distant metastasis, and TNM stage. The high FoxM1 expression also links to a poor prognosis. Moreover, compared with paired adjacent normal tissue, the FoxM1 expression increased significantly in CRC tissue.

## Author contributions

Y.Y. contributed to the design of the study and manuscript writing. X.W., L.J., X.S. contributed to the data extraction and analysis process of the study. S.H. and X.Z. contributed to the financial support and revision of the manuscript.

**Conceptualization:** Yizhou Yao.

**Data curation:** Xuchao Wang, Linhua Jiang, and Xinyu Shao.

**Formal analysis:** Xuchao Wang, Linhua Jiang, and Xinyu Shao.

**Funding acquisition:** Songbing he and Xinguo Zhu.

**Writing – original draft:** Yizhou Yao.

**Writing – review & editing:** Songbing He and Xinguo Zhu.

Songbing He orcid: 0000-0003-4444-6920.
